# Modeling the Effects of Climate Change on Surface Ozone during Summer in the Yangtze River Delta Region, China

**DOI:** 10.3390/ijerph16091528

**Published:** 2019-04-30

**Authors:** Da Gao, Min Xie, Xing Chen, Tijian Wang, Chenchao Zhan, Junyu Ren, Qian Liu

**Affiliations:** 1 School of Atmospheric Sciences, Nanjing University, Nanjing 210023, China; DG1828003@smail.nju.edu.cn (D.G.); xchen@nju.edu.cn (X.C.); tjwang@nju.edu.cn (T.W.); 141170075@smail.nju.edu.cn (C.Z.); tommy_9527@163.com (J.R.); 2 Jiangsu Provincial Academy of Environmental Science, Nanjing 210036, China; antmayiliu@163.com

**Keywords:** climate change, ozone, WRF-Chem, ozone sensitivity

## Abstract

Future climate change can impact ozone concentrations by changing regional meteorological factors related to ozone (O_3_) pollution. To better understand the variations of meteorological factors and their effects on O_3_ formation processes under future climate conditions, we model the present and the future meteorology and air quality in summer over the Yangtze River Delta (YRD) region by using the Weather Research and Forecasting Model with Chemistry module (WRF/Chem), which is driven by the outputs of Community Climate System Model version 4 (CCSM4). The simulations predict that solar radiation, 2-m air temperature, and wind speed increase in the daytime over most of the YRD region. Absolute humidity and precipitation increase in the north and decrease in the south, while the planetary boundary layer height (PBLH) has an opposite change pattern displaying a decrease in the north and an increase in the south. The southerly wind will be strengthened in the daytime. At night, the change patterns of the meteorological factors are similar to the daytime but with small variations. Meanwhile, O_3_ and its precursors all increase in the north and decrease in the south. The increases of NO_x_, volatile organic compounds (VOC), and CO are related with the decreases of PBLH and the input effect of stronger southerly wind, while the decreases are attributed to the output effect of the stronger southerly wind. During the daytime, the increase of surface O_3_ in the north is dominated by the chemical processes related with the increases of solar radiation, air temperature, and O_3_ precursors. The decrease of surface O_3_ in the south is mainly caused by the transport process changing with the strengthened southerly wind. At night, the surface O_3_ changing the amplitude is less than the daytime. The less O_3_ variations at night can be attributed to an O_3_ titration reaction with NO, the changes in NO_x_ concentrations, and the increases of nocturnal PBLH. With the aid of H_2_O_2_/HNO_3_, O_3_ formation in the YRD region is found to be easily affected by NO_x_ in the future. The findings can help to understand the changing trend of O_3_ in the YRD region and can propose reasonable pollution control policies.

## 1. Introduction

At present, climate change and ambient air quality deterioration are serious issues of the atmospheric environment. In the past, they were separately studied by researchers and policymakers. As the research on the relationship between weather, climate, and air pollution deepened, the interaction between climate change and air quality has drawn more concern [1,2,3,4]). Previous studies focused on the impacts of greenhouse gases and aerosols on climate system [5,6], and there are relatively few investigations on the impacts of climate change on ambient air quality [1,4]. The variations of meteorological factors caused by climate change can lead to changes in the air quality [1,4,7,8]. Thus, to better protect our ambient air quality and to adapt ourselves to the changing climate, the impact of regional and global climate changes on ambient air should attract more attention.

Ozone (O_3_), one of the major air pollutants in the air, is harmful to human health and ecological balance [9,10]. In recent years, the regional O_3_ pollution has aggravated. The photochemical reaction is regarded as the main cause of O_3_ pollution, and severe pollution episodes usually occur on sunny days or under adverse weather conditions with plenty of precursors [8,11,12,13,14,15,16,17,18]. Several meteorological factors can affect surface ozone. First, there is a negative correlation between the planetary boundary layer height and ozone concentration [11]. Second, elevated air temperatures can increase the chemical productions of O_3_ and O_3_ concentration [12,13,14,16]. Third, the increased local wind speed can enhance the advection and diffusion processes of O_3_ and thereby abate O_3_ pollution [11]. Moreover, climate change can change these meteorological factors, which may affect the chemical reactions of O_3_ and natural emissions of O_3_ precursors. Consequently, the variation tendency of ground-level O_3_ is inevitably linked with climate change [1,4,7,8]. Over the past few decades, many researchers have studied this issue. Most of them used different models to simulate the O_3_ concentration changes over the next few decades and explored how global climate change would impact regional meteorology and air pollution. Over years of exploration, scholars have gradually realized that the global climate change can affect the emission, transportation, diffusion, chemical reaction, and deposition of air pollutants by affecting the regional air temperature, solar radiation, relative humidity, wind speed, mixed layer height, and emission of ozone precursors [1,4,7,8,11,19,20,21,22,23,24,25,26,27].

With rapid economic development and increasing energy consumption, many regions in China are facing O_3_ pollution [8,15,17,18,28,29,30]. Several researchers linked it with climate change and predicted future O_3_ pollution in China. Based on the simulations from MOZART-2.4, Lin et al. [22] found that O_3_ concentration might increase by 1–5% from 1990 to 2090 in the east of China for the scenario B1 and that the value might be 3–12% for A1FI. Using GEOS-Chem driven by GISS GCM (NASA, Washington, DC, USA), Wang et al. [26] revealed that climate change could increase O_3_ in the east of China and decrease O_3_ in the west of China from 2000 to 2050, and 40% of the increase might result from the strengthened biogenic volatile organic compounds (BVOCs) emission. With the aid of WRF-Chem driven by CCSM3, Liu et al. [7] found that climate change and biogenic emission could result in the increase of O_3_ by 1.6 ppb (−5 to 5 ppb) over South China in 2050, while the uncontrolled anthropogenic emissions would increase O_3_ up to 12.8 ppb. Lee et al. [13] predicted the future O_3_ trend in Hong Kong and indicated that the pollution would continue to rise until 2025. By using WRF-CALGRID (ARB, Sacramento, CA, USA) driven by CCSM3, Xie et al. [8] studied the variation trend of O_3_ in the Yangtze River Delta (YRD) region. They found that the variations in meteorological fields caused by climate change could result in an increase (5 to 15 ppb) in the north and a decrease (−5 to −15 ppb) in the south and that the enhanced natural emissions could contribute approximately 20% to the surface O_3_ increase, with the biggest increase up to 2.4 ppb. Obviously, the trend and the magnitude of O_3_ variations resulted from climate change, varying from region to region in China. Despite a couple of investigations in these dynamic economic regions, more studies are needed. Moreover, among current studies, most researchers only focused on the changes in O_3_ concentration, and few have emphasized the variations of the transportation, diffusion, chemical reaction, and deposition processes of O_3_.

To offer a full understanding of the variation mechanism of O_3_ under the future weather conditions, the air quality model WRF-Chem is applied in this study to quantify the effects of climate change on O_3_ in the YRD—one of the world’s most vigorous economic regions under severe O_3_ pollution. Many surface sites showed that the O_3_ concentration in this region even reached 140 ppb, far above the limit value of 1-hour average O_3_ concentration in the “Ambient air quality standards” (GB3095-2012) published by the Chinese Ministry of Environmental Protection [31,32]. Moreover, the mean monthly highest 5% O_3_ at Lin’an increased at the rate of 1.8 ppb/year during 1996–2006 [33].

As natural emissions and their effects in the YRD under future climate conditions have been studied [8], the impacts of climate change on individual processes of O_3_ formation are discussed in this paper. The remainder of this paper is organized as follows. The model, its configurations, and the input data are introduced in Section 2. The validation of model performance, the change in future meteorology, the impact of climate change on O_3_, its precursors, and O_3_–NO_X_-VOC sensitivity are discussed in Section 3. Finally, a brief summary is given in Section 4.

## 2. Methodology

### 2.1. Model Description and Configuration

In this study, WRF-Chem version 3.5 (NOAA, Colorado, USA) was applied to investigate the impacts of climate change on air quality over the YRD. WRF-Chem is a new generation of air quality modeling system, which is composed of the meteorological component (WRF) and the air quality component (Chem). The two components are fully coupled, and they use the same coordinates and physical parameterizations [34]. WRF-Chem has been widely adopted in simulating the air quality of Chinese city clusters and investigating its formation mechanism [28,29,30,35,36,37]. Also, it is of a relatively good capability in simulating climate change and its effects on air quality [7,8].

**Figure 1:**
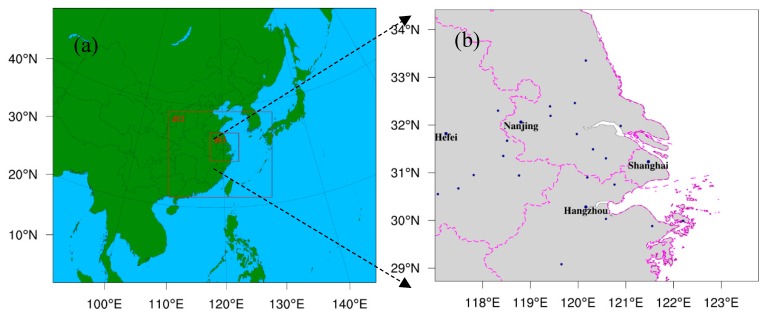
The simulation domain settings in WRF-Chem, including (**a**) the three nested domains and (**b**) the details of domain 3 that mainly covers the Yangtze River Delta (YRD) region.

As shown in Figure 1, three nested domains were used. The model domains were centered at (32.8° N, 87.5° E) with Lambert Conformal Conic projection. The grid sizes of these domains were 56 × 42, 109 × 82, and 178 × 136, with grid spacings of 81, 27, and 9 km, respectively. The outer domain covered a fairly large area of East Asia. The inner domain covered the YRD and its surrounding waters. For all domains, there were 31 vertical layers from the surface to the model top of 100 hPa, with about 10 layers in the planetary boundary layer.

The major physical options selected in WRF-Chem simulations are shown in Table 1. The Purdue Lin microphysics scheme [38] makes progress that the addition of a snow field to the cloud model significantly modifies the microphysical processes; it is more consummate. In the RRTM (Rapid Radiative Transfer Model) longwave radiation scheme, the speed of execution compares favorably with those of other rapid radiation models, and the model can be used in general circulation models [39]. The Goddard shortwave radiation scheme results in considerable improvement in reproducing the model’s thermal structure, such as the zonal mean air temperature, its latitudinal gradient, and vertically integrated temperature [40]. In the Kain–Fritsh cumulus parameterization scheme, the specific formation of the modifications can produce desired effects in numerical weather prediction and render the scheme closer to observations and cloud-resolving modeling studies [41]. The NOAH/LSM (NCAR, Boulder, CO, USA) scheme can not only provide reasonable diurnal variations of surface heat flues but also correct seasonal evolutions of soil moisture in the context of a long-term data assimilation system [42]. The MYJ (Mellor–Yamada–Janjic) PBL scheme makes progress on the basis of the MY PBL scheme, and the new scheme for calculating the MY level 2.5 master length scale is rectified [43]. The chemical mechanism used to simulate gas concentration is the Carbon-Bond Mechanism version Z (CBMZ). The CBMZ is developed from a new lumped-structure mechanism, largely based on the widely used Carbon Bond Mechanism (CBM-IV). Major improvements include a revised inorganic chemistry, an explicit treatment of the lesser reactive paraffins, methane, and ethane [44]. The adopted option for aerosol is the four-bin sectional Model for Simulating Aerosol Interactions and Chemistry (MOSAIC). MOSAIC is found to be in excellent agreement with a benchmark version of the model using a rigorous solver for integrating the stiff ordinary differential equations (ODEs). Therefore, MOSAIC is a good choice in air quality and regional/global aerosol models [45]. The initial and boundary chemical conditions are derived from the modeling results of the global chemistry transport model MOZART-4.

### 2.2. Simulation Cases

To evaluate how climate change influences the O_3_ concentration in the YRD, two simulation cases are specially designed and conducted. One uses the present meteorology in 2014, 2015, and 2016 and the present emission (referred to as PREMET hereafter). The other uses the future meteorology in 2050, 2051, and 2052 and the present emission (referred to as FUTMET hereafter). In the YRD, the observation results show that a high O_3_ concentration usually appears in late spring and early summer and that O_3_ is more sensitive to environment in summer [17,18,29]. Thus, the PREMET simulations are conducted from 00 UTC July 1st to 18 UTC July 31st in 2014, 2015, and 2016. Also, the FUTMET simulations are conducted from 00 UTC July 1st to 18 UTC July 31st in 2050, 2051, and 2052. An initial 48-hour model integration period is used for the spin-up of the simulations. The difference between the modeling results from FUTMET and PREMET can demonstrate the effect of climate change on air quality (EF_climate_), which can be calculated by the following equation:

(1) $${\mathrm{EF}}_{\mathrm{climate}}=\frac{\sum_{t=1}^{N}\left(V_{FUTMET,\;t}-V_{PREMET,\;t}\right)}{N},$$

where *t* represents one modeling time step; *N* represents the total modeled time; $V_{FUTMET,t}$ and $V_{PREMET,t}$ are the hourly modeling outputs of variable *V* (meteorological factors or air pollutants) from FUTMET and PREMET, respectively.

### 2.3. Present and Future Climate Data

The present and future climate data are used to drive WRF as the initial meteorological fields and boundary conditions. These data are obtained from the National Center for Atmospheric Research (NCAR) Community Climate System Model version 4 (CCSM4) outputs with a horizontal resolution of T85 (about 1.41°). The CCSM4 RCP4.5 outputs for 2050, 2051, and 2052 used in this study are based on the Intergovernmental Panel on Climate Change (IPCC) Fifth Assessment Report. RCP4.5 is a midline future climate scenario for CO_2_ emission and economic growth, which can ensure our results to be moderate. For the present year, the CCSM4 outputs in 2014, 2015, and 2016 are used. The data are also compared with the National Centers for Environmental Prediction (NCEP) Final Analysis (FNL) data with the spatial resolution of 1°, and the difference is ignorable.

### 2.4. Anthropogenic Emissions

For the PREMET case, the anthropogenic emissions in China are from the Multi-resolution Emission Inventory (MEIC) developed by Tsinghua University based on a technology-based emission model. The MEIC inventory contains monthly anthropogenic emissions of SO_2_, NO_X_, CO, NH_3_, PM_2.5_, PM_10_, BC, OC, and VOCs in five sectors (agriculture, industry, power plants, residential, and transportation) [46]. Those for the areas out of China are mainly from the inventory developed by the NASA INTEX-B (NASA, Washington, DC, USA), including the emissions of SO_2_, NO_x_, CO, PM_10_, PM_2.5_, BC, OC, and VOCs from the power, residential, industry, and transportation sectors with a resolution of 0.5° [47]. Furthermore, these data have been specially modified for simulations in the YRD following the work of Xie et al. [8,29].

For future anthropogenic emissions, some previous investigations estimated them by some hypothetical growth factors [7,26]. However, the Chinese government has formulated a series of strict emission reduction policies to protect air quality, implying that the hypothetical increase of emissions is inapplicable. On the other hand, the main purpose of this study is to demonstrate the changes of meteorological factors under future climate and their effects on individual processes of O_3_ formation. Therefore, it is reasonable to assume that the future anthropogenic emissions will remain at the current level. In the FUTMET case, the emissions are set to be the same as PREMET.

### 2.5. Process Analysis Method

WRF-Chem version 3.5 contains a simple process analysis function, which can present the contributions of individual atmospheric processes, including chemical reaction (CHEM), vertical mixing coupled with dry deposition (VMIX), and advection transportation with horizontal and vertical components (ADVT). These variables can show the relative significance of each process and provide a particular interpretation of air pollution. In this study, by comparing the values of CHEM, VMIX, and ADVT from FUTMET with those from PREMET, we can figure out how the changes in meteorological factors influence the individual atmospheric processes of O_3_ chemistry. Moreover, the O_3_ change (accumulation or consuming) in different processes is called Chem_O_3_ in the CHEM process, Vmix_O_3_ in the VMIX process, and Advt_O_3_ in the ADVT process. The unit of Chem_O_3_, Vmix_O_3_, and Advt_O_3_ is ppb/h, implying the changes in one hour in this process.

### 2.6. Model Performance Evaluation

The mean bias (MB), root mean square error (RMSE), and correlation coefficient (CORR) between thet observations and the simulation results from PREMET are used to verify the performance of WRF-Chem. In statistics, they are usually defined as

(2) $$MB=\frac{1}{N}\sum_{i=1}^{N}\left(S_{i}-O_{i}\right),$$

(3) $$RMSE=\sqrt{\frac{1}{N}\sum_{i=1}^{N}{\left(S_{i}-O_{i}\right)}^{2}},$$

(4) $$\mathrm{and}\;\mathrm{CORR}=\frac{\sum_{i=1}^{N}\left(S_{i}-S_{m}\right)\left(O_{i}-O_{m}\right)}{\sqrt{\sum_{i=1}^{N}\left(S_{i}-S_{m}\right)}\sqrt{\sum_{i=1}^{N}\left(O_{i}-O_{m}\right)}},$$

where $S_{i}$ and $O_{i}$ are the simulated and the observed values, respectively, and $S_{m}$ and $O_{m}$ are the averaged values of simulation and observation, respectively. Generally, the modeling results are acceptable if MB and RMSE are close to 0 and if CORR is close to 1 [29].

Three observation sites are selected for comparison, namely NJ (32.00° N, 118.80° E) in Nanjing, HZ (30.23° N, 120.16° E) in Hangzhou, and SH (31.40° N, 121.46° E) in Shanghai. Their hourly observational data of 2-m temperature (T_2_), 10-m wind speed (WS_10_), and 2-m relative humidity (RH_2_) in July of 2014, 2015, and 2016 are obtained from the observation database in the Wyoming Weather Web. Their hourly air pollutant concentration records can be acquired from the air quality real-time publishing platform. The observational methods and the quality assurance/quality control (QA/QC) procedures for these data strictly follow the Chinese national standard. Also, the manual inspection of invalid and lacking data is performed during data processing [17,18,28,29,30].

## 3. Results and Discussions

### 3.1. Model Evaluation for WRF-Chem

Table 2 shows the statistical comparisons between the observations and the modeling results from PREMET. OBS and SIM are represented the observation and simulation. For T_2_, the model slightly overvalues T_2_ at all sites, which might result from the uncertainty of urban canopy and surface parameters [28,29,36,37]. However, the overestimation is acceptable because the MB values of T_2_ are only 0.5–1.7 °C, the RMSE of T_2_ is 2.8–3.9 °C, and the CORR between observations and simulations are over 0.8 at all sites (statistically significant at the 95% confidence level). Moreover, the lowest value 0.5 °C for MB and the highest value 0.9 for CORR show that the best simulation of T_2_ is at NJ. For RH_2_, though the modeling results are slightly underestimated, the CORR values are over 0.8 at most sites. Therefore, the simulations for RH_2_ are relatively acceptable. The lowest value 0.7 for CORR is at SH, which may be attributed to the uncertainty of the land-use data that cannot well describe waters around SH [29]. For WS_10_, the modeling values are overestimated at HZ and SH but underestimated at NJ. The CORR (close to 0.5) for WS_10_ is the lowest in three meteorological factors. The wind components were not simulated very well, just as in previous studies, which may be caused by urban canopy parameters [28,29,37]. Compared with previous studies, the biases in this study are acceptable.

With respect to O_3_, the CORRs are more than 0.6 at most sites. The lowest value for MB (−1.2 ppb) and the highest CORR value (close to 0.7) indicate that the simulation at SH is good. However, the modeling results overestimate O_3_ concentrations at NJ and HZ, and the values of MB are around 8.0. These biases might be related to the modeled stronger solar radiation reaching the surface, which can cause positive biases in T_2_ and thereby produces more O_3_ [29,37]. At the same time, the high bias about simulated wind might result in the O_3_ concentration uncertainty. In addition, there are also some uncertainties in the emission, which might cause O_3_ concentration bias.

All in all, the performance of WRF-Chem in modeling climate and air quality is acceptable over the YRD region in this study. Some biases are still found in simulations, but the difference between PREMET and FUTMET can still demonstrate how meteorological factors could impact O_3_, as all other settings are the same in both simulations.

### 3.2. Regional Meteorology Changes

**Figure 2:**
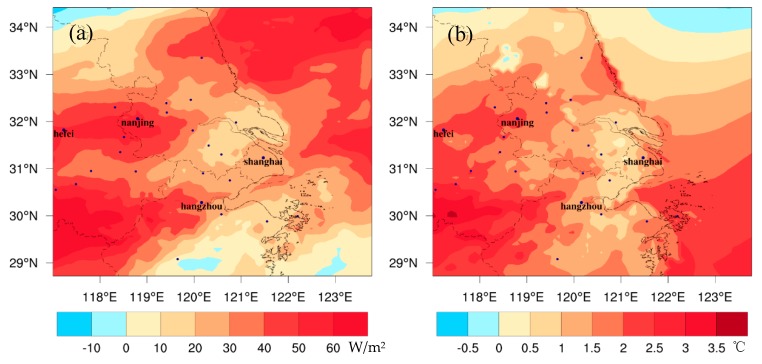

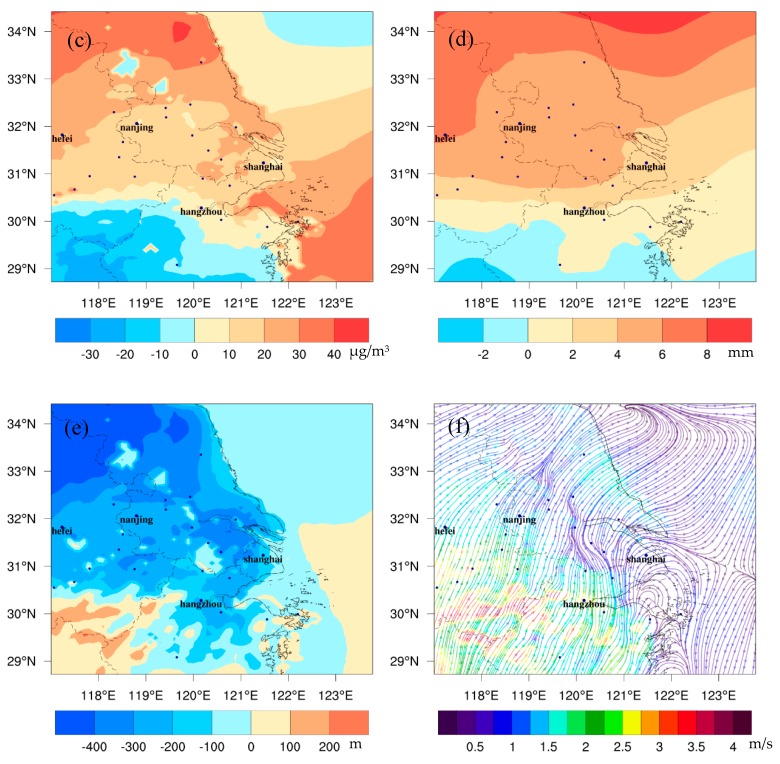
The spatial distribution of the monthly averaged differences of (**a**) solar radiation, (**b**) 2-m air temperature, (**c**) precipitation, (**d**) 2-m absolute humidity, (**e**) planetary boundary layer height, and (**f**) 10-m wind fields between future meteorology in 2050, 2051, and 2052 and the present emission (FUTMET) and the present meteorology in 2014, 2015, and 2016 and the present (PREMET) during the daytime (from 7:00 to 18:00 LST).

**Figure 3:**
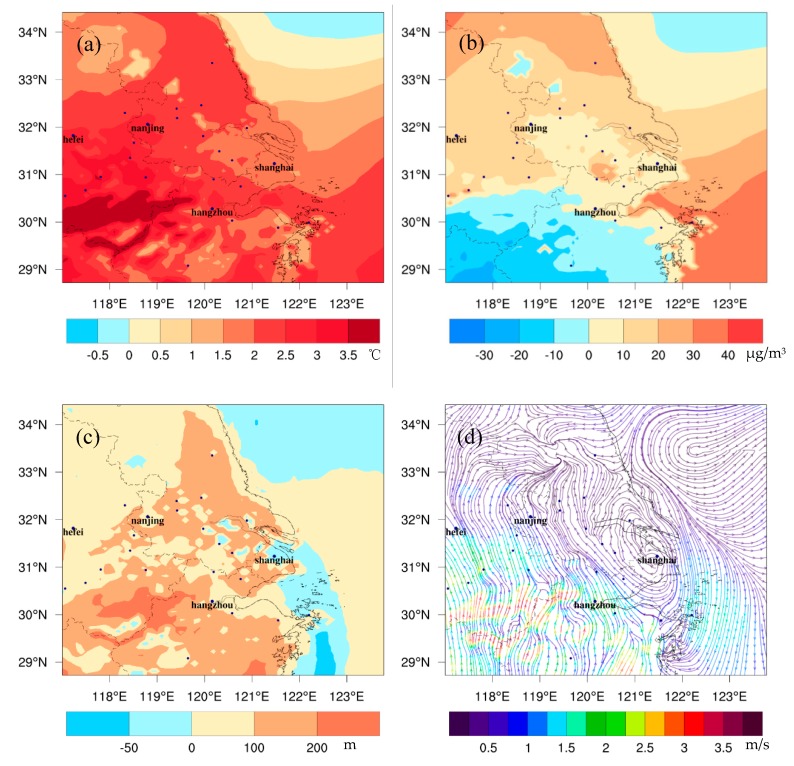
The spatial distribution of the monthly averaged differences of (**a**) 2-m air temperature, (**b**) 2-m absolute humidity, (**c**) planetary boundary layer height, and (**d**) wind fields between FUTMET and PREMET during the nighttime (from 19:00 to 6:00 LST).

The regional meteorological changes caused by global climate change are shown in Figure 2 and Figure 3. The values in these figures present the differences between FUTMET and PREMET. Figure 2 shows the changes during the daytime (from 7:00 to 18:00 LST (Local standard time)). For solar radiation (Figure 2a), the values in the land area of YRD significantly increase, with the average value of 33.2 W/m^2^. The maximum increase of 76.0 W/m^2^ appears in the southwest. The changing pattern of T_2_ (Figure 2b) is similar to that of solar radiation. The average increase of T_2_ is predicted to be 1.5 °C over the entire region. High values can be found in the southwest of the YRD, with a maximum of 3.6 °C. The good relevancy between Figure 2a,b can be explained by the fact that the increase of T_2_ is caused by solar radiation. For absolute humidity (Figure 2c), it increases over 10.0 μg/m^3^ in the northern and central YRD, which is related to the precipitation increase with the value over 4.0 mm in those areas (Figure 2d). However, in the southern YRD, the air becomes dryer (Figure 2c), caused by the precipitation decrease there (Figure 2d). For planetary boundary layer height (PBLH) (Figure 2e), it increases by the maximum value (about 234.3 m) in the southern YRD, which can be attributed to high increments of solar radiation and air temperature. However, PBLH decreases by 200.0 m in most of northern and central areas. The decreases may be caused by the small increase of T_2_ and the high humidity, which can increase latent heat and lower PBLH. For WS_10_ (Figure 2f), the change pattern is similar to that of T_2_. The regional mean increment is 1.2 m/s, and the high increases appear in the south with the maximum value of 4.2 m/s. For the wind direction, a southerly wind will dominate the YRD in the future under the strengthening of the Northwest Pacific Subtropical High.

Figure 3 shows the changes at night (from 19:00 to 6:00 LST). For T_2_ (Figure 3a), it is predicted to increase about 1.9 °C over the entire YRD, with the higher increases over 5.0 °C in the southwest. The increments are slightly higher than those in the daytime, which may be related to more clouds at night. More clouds may strengthen the insulation effect. For absolute humidity (Figure 3b), the regional mean changing value (9.6 μg/m^3^) is smaller than that in the daytime, related to a weaker evaporation at night. For PBLH (Figure 3c), higher increasing values occur in the southwest of the YRD, with a maximum of 268.6 m. The pattern is similar to Figure 3a, implying the effect of air temperature on PBLH. For wind field (Figure 3d), due to the rising of T_2_, the wind speed increases as well. The regional average increase is 0.8 m/s, and the maximum increase is 3.9 m/s. For the wind direction, the southerly wind dominates the region at night just as the changes in the daytime.

The estimated change trends and intensities of the above meteorological factors are in agreement with previous findings [7,8,26]. During the daytime, the increase of solar radiation and T_2_ can enhance the O_3_ photochemical reactions over the entire YRD. Although the increase of PBLH in the north can dilute O_3_ concentration in lower atmosphere, the decrease in the south can increase the O_3_ pollution level. However, the strengthened southerly wind can transport more O_3_ from the south to north, which may increase O_3_ in the north and decrease O_3_ in the south. The change trend of O_3_ in the daytime might be different from that at night. The increase of T_2_ can enhance the titration reaction between NO and O_3_. The increase of PBLH in the north can bring surface O_3_ to the higher layers. The southerly wind may increase O_3_ in the north but decrease it in the south. Consequently, it is hard to tell the change of O_3_ just by the changes in meteorological factors either in the daytime or at night. Further analyses are needed.

### 3.3. Changes in Ozone Precursors

**Figure 4:**
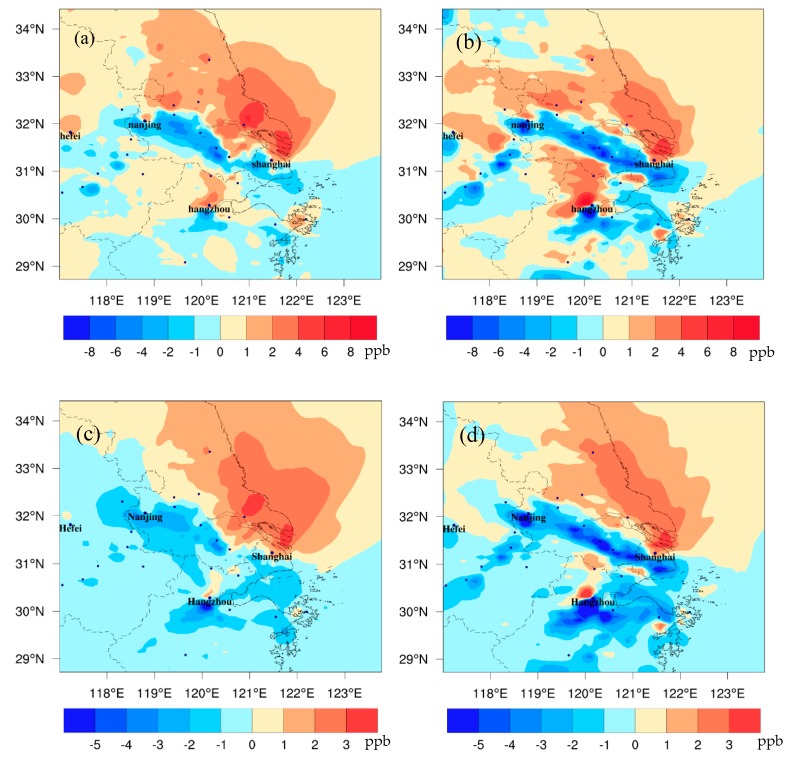

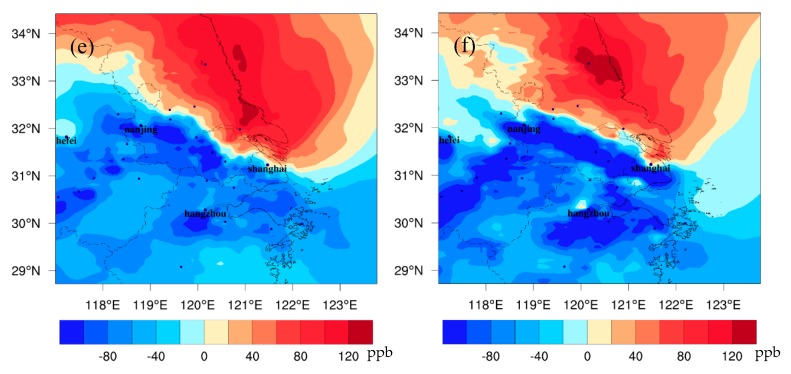
The spatial distribution of the monthly averaged differences of NO_x_ (**a**,**b**), VOC (**c**,**d**), and CO (**e**,**f**) between FUTMET and PREMET. Figure 4a,c,e show the values during the daytime (from 7:00 to 18:00 LST). Figure 4b,d,f present those at night (from 19:00 to 6:00 LST).

Figure 4 shows the differences of ozone precursors at the surface between FUTMET and PREMET. For NO_x_, the change pattern during the daytime (Figure 4a) is similar to that at night (Figure 4b). The increases appear in the north of Shanghai and Hangzhou. In the daytime, the maximum increase is 6.5 ppb in Shanghai. At night, the maximum increase is 11.9 ppb in Hangzhou. These increases of NO_x_ match the decreases of PBLH, implying the accumulation of air pollutants under worse diffusion conditions. Also, the stronger southerly wind in the daytime can lead to increases of concentration in the north. However, more high values appear in Hangzhou at night than in the daytime, caused by an enhancement of the westerly wind along Nanjing-Shanghai line, which blocks the northward transportation of NO_x_. The change patterns of VOCs and CO are similar to that of NO_x_. The higher increments also appear in the northern YRD. The maximum changes for VOCs are 3.7 ppb (Figure 4c) in the day and 6.1 ppb at night (Figure 4d). Those values for CO are 134.3 ppb (Figure 4e) and 154.1 ppb (Figure 4f), respectively [48]. The differences of change patterns between day and night, as well as the main causes, are similar with those of NO_x_.

**Figure 5:**
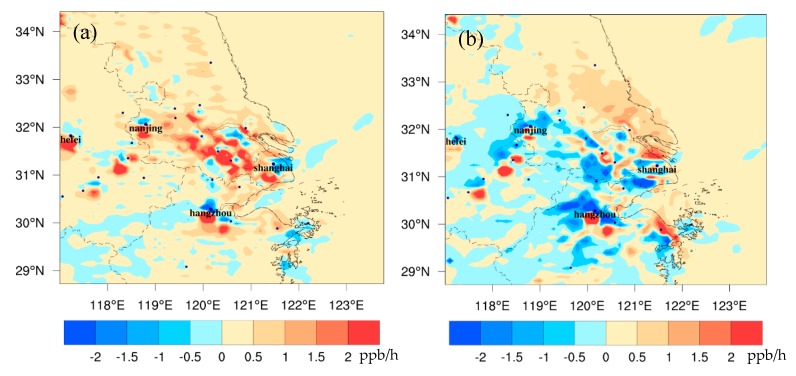

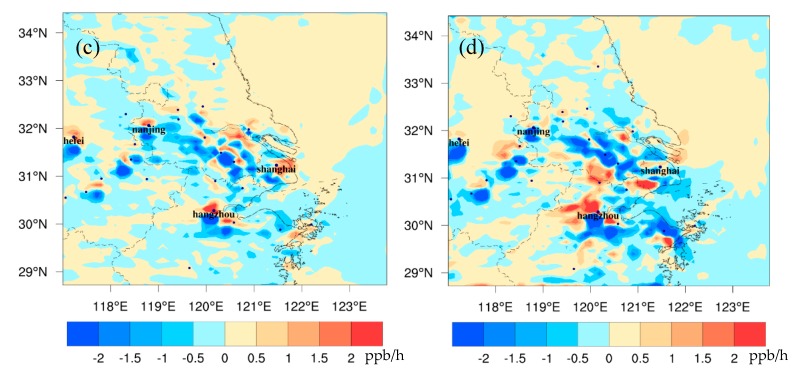
The spatial distribution of the monthly averaged differences of the VMIX processes of NOx Vmix_NO_x_ (**a**,**b**) and the ADVT processes of NOx Advt_NO_x_ (**c**,**d**) between FUTMET and PREMET. Figure 5a,c shows the changes during the daytime (from 7:00 to 18:00 LST). Figure 5b,d present those at night (from 19:00 to 6:00 LST). Vmix_NO_x_ is the contribution of vertical mixing and dry deposition to NO_x_. Advt_NO_x_ is the contribution of horizontal and vertical advection.

To understand the impacts of the changes caused by climate change in individual atmospheric process, the monthly mean differences between FUTMET and PREMET for the CHEM, VMIX, and ADVT processes of NO_x_ in the YRD are investigated. The contributions of VMIX and ADVT to NO_x_ variations are the largest, while CHEM has little effect. Thus, only the changes in the VMIX and ADVT processes of NOx (respectively referred to as Vmix_NOx, and Advt_NOx hereafter) at the surface are presented in Figure 5. As shown in Figure 5a, during the daytime, the maximum increase of Vmix_NO_x_ locates at the center of the YRD with a value of 8.1 ppb/h. This high value should be attributed to the decrease of PBLH (Figure 2e) and higher NO_x_ emissions in this area. At night, however, Vmix_NOx decreases in most of the YRD with a maximum reduction of −7.2 ppb/h. The decreases are related to the increase of PBLH (Figure 3c), which can bring more pollutants to the higher levels. For Advt_NO_x_, its changes are closely related to the variations of wind fields. During the daytime, the increments of Advt_NO_x_ are more than 1.5 ppb/h in the north of the YRD (Figure 5c), implying that the southerly wind (Figure 2f) brings more NO_x_ to the north. At night, NO_x_ is transported from south to north under the dominant southerly wind in most parts (Figure 5d). However, there are high values of Advt_NO_x_ to the south of Nanjing-Shanghai line, which may be connected with a strengthening of the easterly wind in this area in Figure 3d.

### 3.4. Changes in Ozone Driven by Climate Change

**Figure 6:**
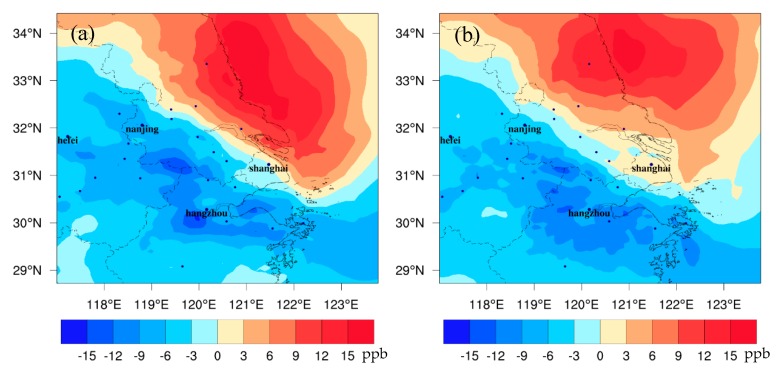
The spatial distribution of the monthly averaged differences of O_3_ between FUTMET and PREMET (**a**) during the daytime (from 7:00 to 18:00 LST) and (**b**) at night (from 19:00 to 6:00 LST).

**Figure 7:**
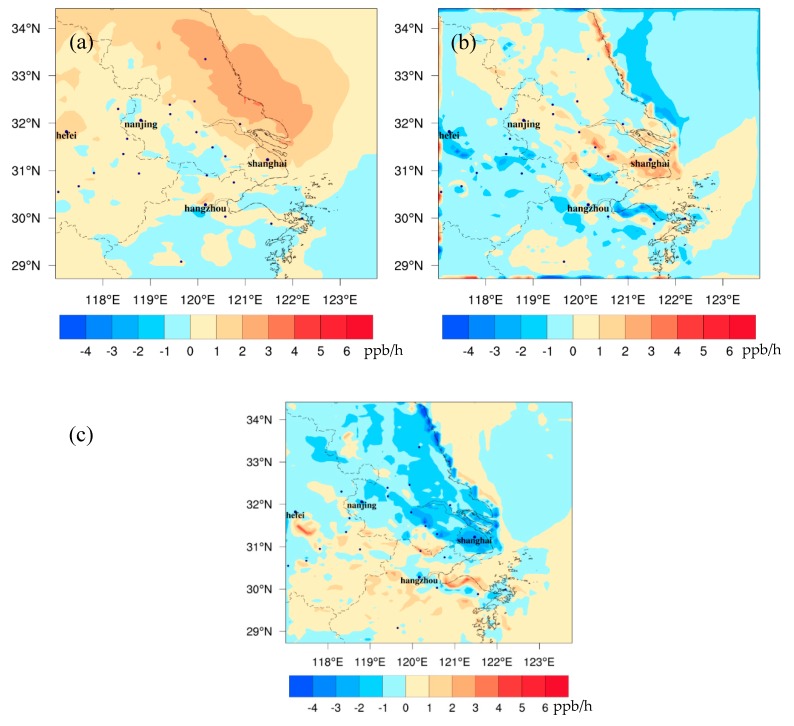
The spatial distribution of monthly averaged differences of (**a**) Chem_O_3_, (**b**) Advt_O_3_, and (**c**) Vmix_O_3_ between FUTMET and PREMET during the daytime (from 7:00 to 18:00 LST). Chem_O_3_ represents the O_3_ chemical production process. Vmix_O_3_ means the contribution of vertical mixing and dry deposition to O_3_. Advt_O_3_ means the contribution of horizontal and vertical advections.

Figure 6 shows the differences of surface ozone between FUTMET and PREMET in the YRD. During the daytime (Figure 7a), O_3_ increases in the north but decreases in the south. The high increase values with a maximum of 18.5 ppb are along the coastal areas of Jiangsu Province, while the high reductions (over −15.1 ppb) are mainly in the inland areas of Jiangsu, Zhejiang, and Anhui Provinces. At night (Figure 7b), the change pattern of O_3_ is similar to that in the day, but the increase and the decrease values are lower. The regional averaged change value is just −0.2 ppb, the maximum increase is 17.5 ppb, and the maximum decrease is about −12.0 ppb. Less O_3_ variations at night may be attributed to nocturnal O_3_ chemical reactions. Moreover, the above O_3_ change patterns are similar to those of NO_x_, VOCs, and CO, implying that O_3_ changes are tightly related to O_3_ precursors and are highly affected by chemical processes.

**Figure 8:**
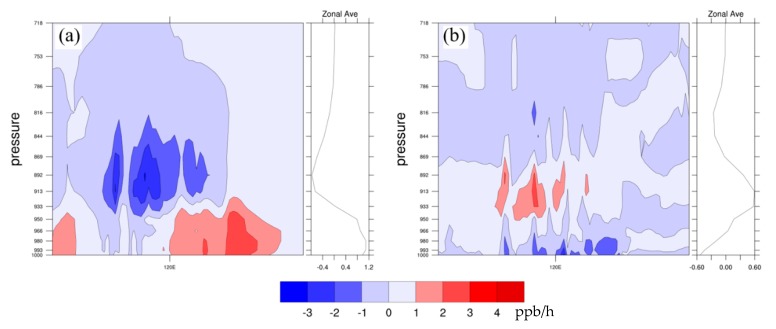
The vertical distribution along 32° N of monthly averaged differences of (**a**) Chem_O_3_ and (**b**) Vmix_O_3_ between FUTMET and PREMET during the daytime (from 7:00 to 18:00 LST). Chem_O_3_ and Vmix_O_3_ represent the chemical production process and the vertical mixing and dry deposition process, rescpectively.

Figure 7 shows the different spatial distributions of major individual O_3_ formation processes between FUTMET and PREMET during the daytime. The changes in O_3_ chemical production (Chem_O_3_) vary from −2 to 4 ppb/h (Figure 7a). The increases of Chem_O_3_ occur in most areas of the YRD, which should be induced by enhanced gas phase reactions related to the increases of solar radiation (Figure 2a) and surface air temperature (Figure 2b). The higher increments over 3.0 ppb/h are mainly located along the coastal areas of Jiangsu Province, which is also the area with great increase of O_3_ precursor concentration (Figure 4a,c,e). Over the southern YRD, the decreases of absolute humidity and O_3_ precursor concentration should result in reductions of Chem_O_3_ in extensive areas, with the maximum decrease about −2.2 ppb/h to the south of Hangzhou. Figure 7b illustrates that the changes in transport process of O_3_ (Advt_O_3_) are between −3.0 to 4.0 ppb/h, which is closely related to the variation of horizontal wind speed and wind direction [7]. Advt_O_3_ rises up over 0–4.0 ppb/h in most areas of the northern YRD, with the higher increments over 2.1 ppb around Shanghai. The decreases of Advt_O_3_ are mainly located in the southern YRD. The change pattern of Advt_O_3_ further proves that the strengthened southerly wind in the YRD (Figure 2f) can bring O_3_ from the south to the north. For the vertical mixing and dry deposition process (Vmix_O_3_), it ranges between –4.0 to 4.0 ppb (Figure 7c). Though PBLH would decrease in the north (Figure 2e, may increase O_3_ at surface), Chem_O_3_ can produce more O_3_ in lower atmospheres (Figure 8a) and more O_3_ would be transported to the upper atmospheric layer or deposition to the ground (Figure 8b). Thus, Vmix_O_3_ in the northern YRD mainly decreases. In the south, however, there is little change in Vmix_O_3_ (0–1.0 ppb/h). The high values close to some cities would be caused by the local atmospheric circulation related to urban heat island [28,29].

**Figure 9:**
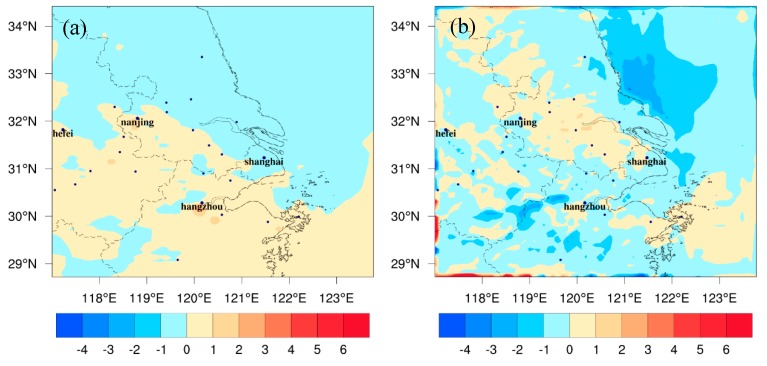

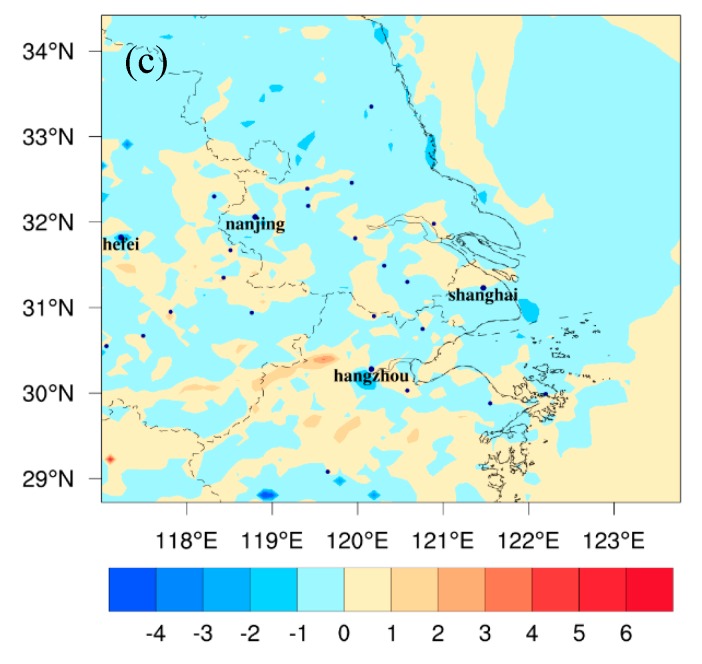
The spatial distribution of monthly averaged differences of (**a**) Chem_O_3_, (**b**) Advt_O_3_, and (**c**) Vmix_O_3_ between FUTMET and PREMET at night (from 19:00 to 06:00 LST). Chem_O_3_ represents the O_3_ chemical production process. Vmix_O_3_ means the contribution of vertical mixing and dry deposition to O_3_. Advt_O_3_ means the contribution of horizontal and vertical advections.

**Figure 10:**
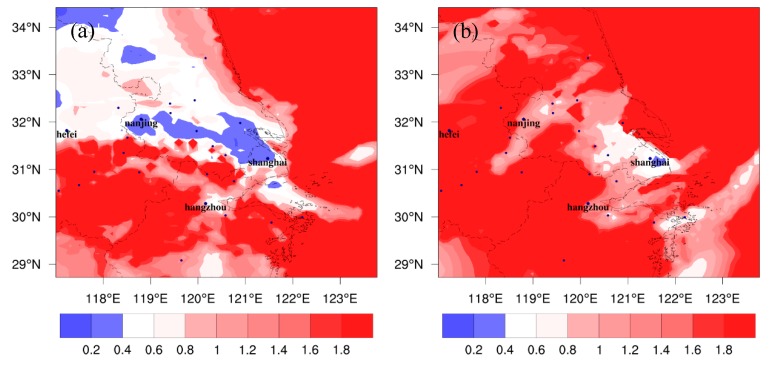
The spatial distributions of monthly mean H_2_O_2_/HNO_3_ during the afternoon (13:00 to 16:00 LST) at the surface over the YRD region under different climate scenarios, including the distribution pattern for (**a**) PREMET and (**b**) FUTMET.

At night, the changes in three main processes are smaller. The changes in Chem_O_3_ vary from −1.0 to 2.0 ppb (Figure 9a). The decreases of Chem_O_3_ mainly appear in the north, which should be attributed to the enhancement of an O_3_ titration reaction with NO at night due to temperature increase (Figure 3a) and the sufficient NO_x_ in the north (Figure 4b). In a similar way, the increases in the south are related with the decrease of NO_x_ there (Figure 4b), which would weaken the O_3_ consumption reaction. Figure 10b shows that the changes in Advt_O_3_ are between −3.0 and 2.0 ppb in most areas. O_3_ accumulation along the Nanjing-Shanghai line is due to the strengthening of the easterly wind. The changes in Vmix_O_3_ range between −3.0 and 2.0 ppb (Figure 9c). The decreases of Vmix_O_3_ are related to the increases of nocturnal PBLH over the YRD (Figure 3c). The maximum increases of Vmix_O_3_ are in the south of the YRD. The increases are caused by great increases of wind speed that can weaken the dry deposition in this area (Figure 3d).

### 3.5. Impact of Climate Change on Regional Ozone Control Policy

The ratio of certain secondary photochemical products, such as H_2_O_2_/NO_Z_ and H_2_O_2_/HNO_3_, can be used as the efficient indicators to distinguish NO_X_- or VOC-sensitive regimes of O_3_ chemistry (Xie et al., 2014). In this study, H_2_O_2_/HNO_3_ is adopted to investigate the O_3_–NO_X_-VOC sensitivity in the YRD under different climate conditions. According to the previous study [8], the transition value of H_2_O_2_/HNO_3_ in the present summer is quantified to be 0.3–0.5, while it will change to 0.4–0.8 under the future conditions.

Figure 10 shows the mean values of H_2_O_2_/HNO_3_ at the first model layer over the YRD during afternoon (13:00–16:00 LST) from FUTMET and PREMET. For PREMET, low values (<0.3) of H_2_O_2_/HNO_3_ are mainly located in Shanghai, Nanjing, Hangzhou, and most land areas in the northern YRD (Figure 10a). The results mean that the O_3_ chemistry in the typical cities and the northern YRD is VOC-sensitive and that VOCs should be preferentially reduced in these areas at present. However, in the future, more and more areas in YRD are covered by high values of H_2_O_2_/HNO_3_ (Figure 10b). The values in the vast majority of grids are over 0.8, which means the O_3_ chemistry is NO_x_-sensitive in most parts of the YRD. Lower values (<0.4) only appear around Shanghai. The values in the cities of southern Jiangsu and northeastern Zhejiang are also close to 0.4. The change in the distribution pattern of H_2_O_2_/HNO_3_ implies that O_3_ chemistry in the future of YRD tends to be insensitive to VOCs and is more easily affected by NO_x_, which is in accordance with the findings of Xie et al. [8]

## 4. Conclusions

The effects of climate change on surface ozone in summer over the YRD region is studied by using WRF-Chem, with a special emphasis on the changes in meteorological factors and their impacts on individual atmospheric processes of O_3_ formation.

The simulations predict that solar radiation and a 2-m air temperature increase in the daytime in most of the YRD region with average increments of 33.2 W/m^2^ and 1.5 °C, respectively. The absolute humidity and precipitation increase in the north and decrease in the south with variations of −30.0–40.0 μg/m^3^ and −2.0–8.0 mm, respectively. PBLH increases about 100.0 to 200.0 m in the south and decrease about −100.0 to −400.0 m in the north. The change pattern of wind speed is similar to air temperature, with an average increment of 1.2 m/s. The southerly wind will be strengthened. At night, the change patterns of the meteorological factors are similar to those during the daytime but with small variations. The regional average increments of T_2_ and wind speed are 1.9 °C and 0.8 m/s, respectively. Easterly wind will be strengthened along the Shanghai-Nanjing line. The absolute humidity varies −20.0 to 30.0 μg/m^3^, and PBLH increases about 100.0–200.0 m.

For the effects of climate change on air pollutants, the change patterns of O_3_ precursors (NO_x_, VOC, and CO) are similar, with an increase in the north and a decrease in the south. The maximum increments of NO_x_, VOC, and CO in the day can reach 6.5, 3.7, and 134.3 ppb, respectively. Those at night are 11.9, 6.1, and 154.1 ppb, respectively. According to a process analysis, their increases are related with the decreases of PBLH and the input effect of stronger southerly wind, while the decreases are attributed to the output effect of the stronger southerly wind. Surface O_3_ variations will increase in the north and decrease in the south during the daytime. According to process analysis, the increase of surface O_3_ in the north is dominated by gas phase chemical process related to the increases of solar radiation, air temperature, and O_3_ precursors. The decrease in the south is mainly caused by the transport process changing with the strengthened southerly wind. During the nighttime under the future climate, the surface O_3_ changes amplitude is less than the daytime, with the same change pattern as that in the day. The less O_3_ variations at night can be attributed to an O_3_ titration reaction with NO, the changes in NO_x_ concentrations, and the increases of nocturnal PBLH. Moreover, climate change can affect the chemical relationship between O_3_ and its precursors. With the aid of H_2_O_2_/HNO_3_, O_3_–NO_x_-VOC sensitivity over the YRD region is found to be easily affected by NO_x_ in the future.

This study provides us a scope of understanding how the future climate affects the surface ozone in the YRD region. However, only the IPCC RCP4.5 scenario from CCSM4 is considered. To obtain a comprehensive understanding, the future meteorological inputs should be provided by more global climate models based on more future climate scenarios, including Socio-Economic Pathways (SSPs) scenarios. Moreover, the manmade emissions may decrease in the future, and the land-use types may continue to change for decades. These future changes should be taken into consideration in the follow-up studies as well.

## Figures and Tables

**Table 1:** The grid setting, physics, and chemistry options used in WRF-Chem simulations.

Items	Contents
Dimensions (x, y)	(85, 750), (76, 70), (76, 70)
Grid size (km)	81, 27, 9
Time step (s)	360
Microphysics	Purdue Lin microphysics scheme
Long wave radiation	Rapid Radiative Transfer Model (RRTM) scheme
Shortwave radiation	Goddard scheme
Cumulus parameterization	Kain–Fritsch scheme, only for D01 and D02
Land surface	NOAH land surface model
Planetary boundary layer	Mellor–Yamada–Janjic scheme
Gaseous chemical mechanism	Carbon-Bond Mechanism version Z (CBMZ)
Aerosol module	Model for Simulating Aerosol Interactions and Chemistry (MOSAIC) using 8 sectional aerosol bins

**Table 2:** The statistics of meteorological conditions and ozone concentrations from PREMET at three sites.

Vars	Sites	OBS	SIM	MB	RMSE	CORR
T2 (°C)	NJ	26.9	27.4	0.5	3.9	0.9
	HZ	28.2	30.0	1.7	3.4	0.9
	SH	27.6	28.5	0.8	2.8	0.8
RH2 (%)	NJ	82.4	81.5	−0.9	14.0	0.9
	HZ	76.9	72.0	−4.9	13.3	0.8
	SH	78.7	75.2	−3.5	11.0	0.7
WS10 (m/s)	NJ	2.3	2.0	−0.2	1.2	0.6
	HZ	2.0	2.0	0.1	1.1	0.5
	SH	1.1	3.5	2.5	1.8	0.4
O3 (ppb)	NJ	35.9	43.5	7.6	23.0	0.6
	HZ	35.6	43.5	7.9	23.6	0.6
	SH	33.5	32.3	−1.2	32.2	0.7

OSB: observation, SIM: simulation, MB: mean bias, RMSE: root mean square error; CORR: correlation coefficient.

## References

[1] Jacob D.J., Winner D.A. (2009). Effect of climate change on air quality. Atmos. Environ..

[2] Athanassiadou M., Baker J., Carruthers D., Collins W., Girnary S., Hassell D., Hort M., Johnson C., Johnson K., Jones R. (2010). An assessment of the impact of climate change on air quality at two UK sites. Atmos. Environ..

[3] Hassan N.A., Hashim Z., Hashim J.H. (2016). Impact of Climate Change on Air Quality and Public Health in Urban Areas. Asia-Pac. J. Public Health.

[4] Von Schneidemesser E., Monks P.S., Allan J., Bruhwiler L., Forster P., Fowler D., Lauer A., Morgan W.T., Paasonen P., Righi M. (2015). Chemistry and the Linkages between Air Quality and Climate Change. Chem. Rev..

[5] Paeth H., Feichter J. (2006). Greenhouse-gas versus aerosol forcing and African climate response. Clim. Dyn..

[6] Kodros J.K., Scott C.E., Farina S.C., Lee Y.H., L’Orange C., Volckens J., Pierce J., L’orange C. (2015). Uncertainties in global aerosols and climate effects due to biofuel emissions. Atmos. Chem. Phys. Discuss..

[7] Liu Q., Lam K., Jiang F., Wang T., Xie M., Zhuang B., Jiang X. (2013). A numerical study of the impact of climate and emission changes on surface ozone over South China in autumn time in 2000–2050. Atmos. Environ..

[8] Xie M., Shu L., Wang T.-J., Liu Q., Gao D., Li S., Zhuang B.-L., Han Y., Li M.-M., Chen P.-L. (2017). Natural emissions under future climate condition and their effects on surface ozone in the Yangtze River Delta region, China. Atmos. Environ..

[9] Wittig V.E., Ainsworth E.A., Long S.P. (2007). To what extent do current and projected increases in surface ozone affect photosynthesis and stomatal conductance of trees? A meta-analytic review of the last 3 decades of experiments. Plant Cell.

[10] Ainsworth E.A., Yendrek C.R., Sitch S., Collins W.J., Emberson L.D. (2012). The Effects of Tropospheric Ozone on Net Primary Productivity and Implications for Climate Change. Annu. Rev. Plant Biol..

[11] Sanchez-Ccoyllo O.R., Ynoue R.Y., Martins L.D., Andrade M.D. (2006). Impacts of ozone precursor limitation and meteorological variables on ozone concentration in Sao Paulo, Brazil. Atmos. Environ..

[12] Stathopoulou E., Mihalakakou G., Santamouris M., Bagiorgas H.S. (2008). On the impact of temperature on tropospheric ozone concentration levels in urban environments. J. Earth Sci..

[13] Lee Y.C., Shindell D.T., Faluvegi G., Wenig M., Lam Y.F.N., Ning Z., Hao S., Lai C.S. (2014). Increase of ozone concentrations, its temperature sensitivity and the precursor factor in South China. Tellus B Chem. Phys. Meteorol..

[14] Fu T.M., Zheng Y.Q., Paulot F., Mao J.Q., Yantosca R.M. (2015). Positive but variable sensitivity of August surface ozone to large-scale warming in the southeast United States. Nat. Clim. Chang..

[15] Xie M., Zhu K., Wang T., Yang H., Zhuang B., Li S., Li M., Zhu X., Ouyang Y. (2014). Application of photochemical indicators to evaluate ozone nonlinear chemistry and pollution control countermeasure in China. Atmos. Environ..

[16] Delcloo A.W., Duchêne F., Hamdi R., Berckmans J., Deckmyn A., Termonia P. (2016). The Impact of Heat Waves and Urban Heat Island on the Production of Ozone Concentrations Under Present and Future Climate Conditions for the Belgian Domain. International Technical Meeting on Air Pollution Modelling and Its Applicatio.

[17] Xie M., Zhu K., Wang T., Chen P., Han Y., Li S., Zhuang B., Shu L. (2016). Temporal characterization and regional contribution to O_3_ and NOx at an urban and a suburban site in Nanjing, China. Sci. Total Environ..

[18] Shu L., Xie M., Wang T., Gao D., Chen P., Han Y., Li S., Zhuang B., Li M. (2016). Integrated studies of a regional ozone pollution synthetically affected by subtropical high and typhoon system in the Yangtze River Delta region, China. Atmos. Chem. Phys. Discuss..

[19] Tagaris E., Manomaiphiboon K., Liao K.-J., Leung L.R., Woo J.-H., He S., Amar P., Russell A.G. (2007). Impacts of global climate change and emissions on regional ozone and fine particulate matter concentrations over the United States. J. Geophys. Res. Biogeosci..

[20] Mahmud A., Tyree M., Cayan D., Motallebi N., Kleeman M.J. (2008). Statistical downscaling of climate change impacts on ozone concentrations in California. J. Geophys. Res. Atmos..

[21] Kovac-Andric E., Brana J., Gvozdic V. (2009). Impact of meteorological factors on ozone concentrations modelled by time series analysis and multivariate statistical methods. Ecol. Inform..

[22] Lin J.T., Patten K.O., Hayhoe K., Liang X.Z., Wuebbles D.J. (2009). Effects of future climate and biogenic emissions changes on surface ozone over the united states and china. J. Appl. Meteorol. Climatol..

[23] Lam Y.F.N., Fu J.S., Wu S., Mickley L.J. (2011). Impacts of future climate change and effects of biogenic emissions on surface ozone and particulate matter concentrations in the United States. Atmos. Chem. Phys. Discuss..

[24] Im U., Poupkou A., Incecik S., Markakis K., Kindap T., Melas D., Yenigun O., Topcu S., Odman M.T., Tayanç M. (2011). The Impact of Anthropogenic and Biogenic Emissions on Surface Ozone Concentrations in Istanbul. Sci Total Environ..

[25] Gao Y., Fu J.S., Drake J.B., Lamarque J.-F., Liu Y. (2013). The impact of emissions and climate change on ozone in the United States under Representative Concentration Pathways (RCPs). Atmos. Chem. Phys. Discuss..

[26] Wang Y.X., Shen L., Wu S.L., Mickley L., He J.W., Hao J.M. (2013). Sensitivity of surface ozone over China to 2000-2050 global changes of climate and emissions. Atmos. Environ..

[27] Henneman L.R., Chang H.H., Liao K.J., Lavoué D., Mulholland J.A., Russell A.G. (2017). Accountability assessment of regulatory impacts on ozone and PM2.5 concentrations using statistical and deterministic pollutant sensitivities. Air Qual. Atmos. Health.

[28] Xie M., Zhu K., Wang T., Wen F., Da G., Li M., Li S., Zhuang B., Han Y., Chen P. (2016). Changes in regional meteorology induced by anthropogenic heat and their impacts on air quality in South China. Atmos Chem Phys..

[29] Xie M., Liao J., Wang T., Zhu K., Zhuang B., Han Y., Li M., Li S. (2016). Modeling of the anthropogenic heat flux and its effect on regional meteorology and air quality over the Yangtze River Delta region, China. Atmos. Chem. Phys. Discuss..

[30] Zhu K.G., Xie M., Wang T.J., Cai J.X., Li S.B., Feng W. (2017). A modeling study on the effect of urban land surface forcing to regional meteorology and air quality over South China. Atmos. Environ..

[31] Li M.M., Wang T.J., Xie M., Zhuang B.L., Li S., Han Y., Song Y., Cheng N.L. (2017). Improved meteorology and ozone air quality simulations using MODIS land surface parameters in the Yangtze River Delta urban cluster, China. J. Geophys. Res-Atmos..

[32] She Q.N., Peng X., Xu Q., Long L.B., Wei N., Liu M., Jia W.X., Zhou T.Y., Han J., Xiang W.N. (2017). Air quality and its response to satellite-derived urban form in the Yangtze River Delta, China. Ecol Indic.

[33] Xu X., Lin W., Wang T., Yan P., Tang J., Meng Z., Wang Y. (2008). Long-term trend of surface ozone at a regional background station in eastern China 1991–2006: Enhanced variability. Atmos. Chem. Phys..

[34] Grell G.A., Peckham S.E., Schmitz R., McKeen S.A., Frost G., Skamarock W.C., Eder B. (2005). Fully coupled “online” chemistry within the WRF model. Atmos. Environ..

[35] Yu M., Carmichael G.R., Zhu T., Cheng Y. (2014). Sensitivity of predicted pollutant levels to anthropogenic heat emissions in Beijing. Atmos. Environ..

[36] Liao J.B., Wang T.J., Wang X.M., Xie M., Jiang Z.Q., Huang X.X., Zhu J.L. (2014). Impacts of different urban canopy schemes in WRF/Chem on regional climate and air quality in Yangtze River Delta, China. Atmos. Res..

[37] Liao J.B., Wang T.J., Jiang Z.Q., Zhuang B.L., Xie M., Yin C.Q., Wang X.M., Zhu J.L., Fu Y., Zhang Y. (2015). WRF/Chem modeling of the impacts of urban expansion on regional climate and air pollutants in Yangtze River Delta, China. Atmos Environ..

[38] Lin Y.L., Farley R.D., Orville H.D. (1983). Bulk Parameterization Of the Snow Field In a Cloud Model. J. Clim. Appl. Meteorol..

[39] Mlawer E.J., Taubman S.J., Brown P.D., Iacono M.J., Clough S.A. (1997). Radiative transfer for inhomogeneous atmospheres: RRTM, a validated correlated-k model for the longwave. J. Geophys. Res. Biogeosci..

[40] Kim H.J., Wang B. (2011). Sensitivity of the WRF Model Simulation of the East Asian Summer Monsoon in 1993 to Shortwave Radiation Schemes and Ozone Absorption. Asia-Pac. J. Atmos. Sci..

[41] Kain J.S. (2004). The Kain–Fritsch Convective Parameterization: An Update. J. Appl. Meteorol..

[42] Chen F., Dudhia J. (2001). Coupling an Advanced Land Surface–Hydrology Model with the Penn State–NCAR MM5 Modeling System. Part I: Model Implementation and Sensitivity. Mon. Weather. Rev..

[43] Janjic Z.I. (1994). The Step-Mountain Eta Coordinate Model—Further Developments of the Convection, Viscous Sublayer, And Turbulence Closure Schemes. Mon. Weather Rev..

[44] Zaveri R.A., Peters L.K. (1999). A new lumped structure photochemical mechanism for large-scale applications. J. Geophys. Res. Biogeosci..

[45] Zaveri R.A., Easter R.C., Fast J.D., Peters L.K. (2008). Model for Simulating Aerosol Interactions and Chemistry (MOSAIC). J. Geophys. Res. Biogeosci..

[46] Lei Y., Zhang Q., He K.B., Streets D.G. (2011). Primary anthropogenic aerosol emission trends for China, 1990–2005. Atmos. Chem. Phys. Discuss..

[47] Zhang Q., Streets D.G., Carmichael G.R., He K.B., Huo H., Kannari A., Klimont Z., Park I.S., Reddy S., Fu J.S. (2009). Asian emissions in 2006 for the NASA INTEX-B mission. Atmos. Chem. Phys. Discuss..

[48] Hang J., Luo Z., Wang X., He L., Wang B., Zhu W. (2016). The influence of street layouts and viaduct settings on daily carbon monoxide exposure and intake fraction in idealized urban canyons. Environ. Pollut..

